# Detecting reperfusion myocardial hemorrhage with T2 and T2* maps at 1.5T

**DOI:** 10.1186/1532-429X-13-S1-P112

**Published:** 2011-02-02

**Authors:** Avinash Kali, Andreas Kumar, Xiangzhi Zhou, Veronica LM Rundell, Ying Liu, Rachel A Klein, Richard LQ Tang, Rohan Dharmakumar

**Affiliations:** 1 Northwestern University, Chicago, IL, USA; 2 Laval Univeristy, Quebec, QC, Canada

## Introduction

Reperfusion into severely ischemic myocardium can lead to myocardial hemorrhage (MH). Recent studies have employed T2- and T2*-weighted imaging to assess MH. However, a direct comparison of myocardial T2 and T2* changes in the setting of MH is not available.

## Purpose

To examine the T2 and T2* changes associated with MH and thereby determine the effectiveness of each method for detecting MH.

## Methods

Canines (n = 9) were subjected to a 3-hour occlusion of the LAD followed by reperfusion. Serial CMR studies (1.5T Siemens Espree) were performed post-reperfusion on days 2, 5 and 7. Short-axis images of the entire LV (resolution = 1.1x1.1x8mm3) were obtained using T2-prepared SSFP (T2-preparation= 0, 24 and 55ms), multi gradient-echo (TE=3.43ms, 6.42ms, 9.41ms, 12.40ms, 15.39ms and 18.38ms) and PSIR Late-Enhancement (LE-PSIR) imaging. T2 and T2* maps were computed from T2-prepared and gradient-echo acquisitions. Hemorrhagic infarctions (MH+) were determined by the presence of hypointense territories on T2* maps within the infarcted zones identified from LE-PSIR images. In the MH+ group, manually drawn ROIs on the T2* maps around the hemorrhagic cores and remote territories were copied to the T2 maps. In non-hemorrhagic infarctions (MH-), manually drawn ROIs on LE-PSIR images around the infarcted zones and remote territories were copied to T2 and T2* maps. T2 and T2* values from the MH+, MH- and remote territories were measured and compared (p<0.05).

## Results

**Figure 1:**
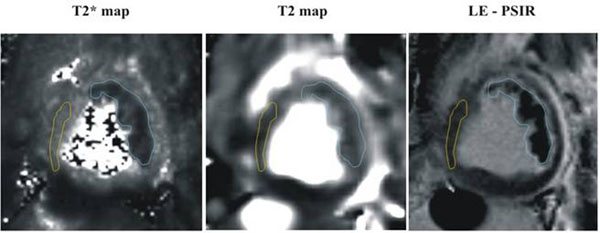
T2* map, TW map and Late-Enhancement (LE) PSIR image from an LAD instrumented canine 5 days post reperfusion are shown. ROIs were manually traced around the hemorrhagic cores (represented here with a blue boundary) on the basis of T2* maps, and remote territories (yellow boundary) on the basis of L-PSIR images. As evident from the images, T2* changes with respect to remote territories were more pronounced than the T2 changes in the presence of hemorrhage.

**Figure 2:**
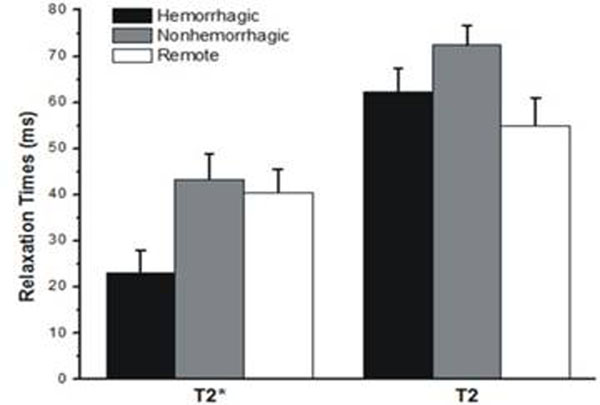
Mean T2* and T2 values for hemorrhagic (black), non-hemorrhagic (gray) and remote territories (white) are shown. T2* values in the presence of hemorrhage were significantly lower than the non-hemorrhagic infarctions and remote territories. T2 values of hemorrhagic infarctions were not significantly different from those of the remote territories. However, the T2 values of non-hemorrhagic infarctions were significantly higher than the hemorrhagic and remote territories.

MH was observed in 6 dogs (MH+), but not in the remaining 3 dogs (MH-). Figure 1 shows a representative set of T2* and T2 maps and the corresponding LE image in a canine with MH. Figure 2 shows the mean T2* and T2 values from MH+, MH-, and remote territories. Table 1 lists the respective relaxation values and the change in T2 and T2* between MH+, MH-, and remote territories. In the MH+ group, T2* values decreased by 46% compared to the MH- group, while T2 decreased by only 16% (p<0.05).

**Table 1:** Mean T2* and T2 values from hemorrhagic, non-hemorrhagic and remote territories were computed by averaging across all the days from all the animals

Region	Remote Myocardium	Hemorrhagic infarction (MH+)	Non-hemorrhagic infarction (MH-)	% Change with respect to remote myocardium
Technique				MH+	MH-
T2* (ms)	40.5 ± 5.0	23.0 ± 4.9	43.3 ± 5.6	-42.1 ± 14.1%*	7.9 ± 17.3%
**T2 (ms)**	54.9 ± 6.0	62.3 ± 5.1	72.5 ± 4.3	12.8 ± 14.4 %	35/3 ± 11.3%*

Mean percentage changes of T2* and T2 values in hemorrhagic and nom-hemorrhagic infarctions with respect to remote territories are shown. Statistically significant changers (p<0.05) are denoted with an asterisk (*). While there was a significant decrease in T2* in hemorrhagic infarctions with respect to remote territories, T2 changes were insignificant. In non-hemorrhagic infarctions T2 was significantly higher, while T2* change was insignificant.

## Conclusion

T2* of MH+ territories were significantly lower than those of MH- and remote territories. This was not the case amongst T2 of MH+, MH- and remote territories. The reduced conspicuity of MH on T2 maps is likely due to the refocusing effects of the 180o pulses and its intrinsic sensitivity to myocardial edema. We conclude that the potential insensitivity of T2*-weighted CMR to myocardial edema and strong sensitivity to hemorrhagic byproducts makes T2* mapping a more effective approach for detecting MH.

